# Prosthodontic Rehabilitation of Arabic Speaking Individuals with Velopharyngeal Incompetence: A Preliminary Study

**DOI:** 10.2174/1874210601711010436

**Published:** 2017-08-30

**Authors:** Abdel Rahim M. Bibars, Firas S.D. Alfwaress, Abed Al-Hadi Hamasha, Zeid A. Al-Hourani, Khader Almhdawi

**Affiliations:** 1 Department of Applied Dental Sciences, Faculty of Applied Medical Sciences, Jordan University of Science and Technology, Irbid 22110, Jordan; 2 Department of Rehabilitation Sciences, Faculty of Applied Medical Sciences; Jordan University of Science and Technology, Irbid, Jordan; 3 Department of Preventive Dentistry, Faculty of Dentistry, Jordan University of Science and Technology, Irbid, Jordan

**Keywords:** Velopharyngeal impairment, Velopharyngeal incompetence, Velopharyngeal insufficiency PLA, Palatal lift prosthesis, Prosthodontic rehabilitation, Velopharyngeal valve

## Abstract

**Background:**

Hypernasality is a frequently encountered problem in the speech of individuals with velopharyngeal incompetence. The use of palatal lift appliance (PLA) is the main treatment option for correction of velopharyngeal incompetence. The literature on the outcomes of using prosthetics treatment for Arabic speaking patients is scarce.

**Objective:**

The aim of this study was to investigate the effect of using PLA on hypernasality of Arabic speaking patients with velopharyngeal incompetence.

**Methods:**

Six participants with age ranging from 9 to 61 years (4 males and 2 females) were recruited between October 2013 and August 2014. Written informed consents were taken from all the adult participants/the guardians of under-aged participants. All patients exhibited hypernasality with different etiologies for velopharyngeal incompetence (head injury, cerebrovascular accident, and neurological disorders). They were treated with PLAs which were constructed to elevate the dysfunctional soft palate. Nasalance scores and perceptual speech acceptability ratings were measured/evaluated in both situations; with and without appliances. Paired t-test was used to analyze the perceptual ratings and nasalance scores in order to detect any significant change in hypernasality pre and post insertion of PLA.

**Results:**

There was a statistically significant decrease (*p*>0.05) in nasalance scores (Pa, Pi, Ma, Mi, a, i) after PLA insertion. The subtest /u/ showed insignificant change (*p*= 0.056). Perceptual ratings showed significant reduction in hypernasality which was consistent with nasalance measurements.

**Conclusion:**

PLAs can reduce hypernasality in Arabic speaking patients who suffer from velopharyngeal impairment.

## 1 INTRODUCTION

The velopharyngeal (VP) valve is a tridimensional muscular valve which is situated posteriorly between the oral and nasal cavities. It consists of the soft palate and pharyngeal walls which regulate resonation and speech utterances as well as other non-speech functions such as swallowing, whistling, blowing, and sucking [1, 2].

During the production of oral sounds, the soft palate elevates and retracts towards the posterior pharyngeal wall whereas it is lowered during the production of nasal sounds. The synergetic behavior of pharyngeal and buccal muscles generates a type of constriction called velopharyngeal closure [3]. During non-speech functions such as swallowing, the soft palate separates the nasal cavity at the level of oropharynx, hence avoid food entering into the nasal cavity [4].

Velopharyngeal impairment may occur when this valve is incapable to perform its closure because of VP insufficiency or VP incompetency [5]. This impairment may impact the oral and nasal coupling which leads to many clinical implications such as problems with resonance and speech intelligibility as well as deglutition and swallowing [6]. Correction of velopharyngeal impairment is considered a multidisciplinary approach involving the speech pathologist, prosthodontist, maxillofacial surgeon, and dental technologist [7].

VP insufficiency is defined as the failure to close the valve due to lack of tissue, *i.e.*, soft palate becomes short. On the other hand, VP incompetence results from poor coordination and timing of velopharyngeal movement.

In both the conditions, the intraoral pressure is reduced due to the leakage of the air flow into the nasal cavity rather than being used for the production of oral sounds. This leakage affects the production of oral sounds drastically especially the production of pressure consonants.

Palatal lift appliance (PLA) is a special appliance reserved for patients in whom there are sufficient tissues but experience poor control of timing and coordination of VP movement [8, 9]. The aim of using PLA is to improve resonance imbalance by repositioning the soft palate to its assumed level of elevation, therefore, closing the velopharyngeal valve to eliminate hypernasality and nasal emission during the production of oral consonants [10, 11]. Many studies showed that prosthetic treatment can provide patients with a socially acceptable speech that help them overcoming the deficiency [5]. There is a lack of literature investigating the effect of prosthetic management on hypernasality and excessive nasal airflow among Arabic speaking individuals. Therefore, the present study aims to 1) describe the fabrication of PLA and 2) highlight the effectiveness of PLA in correcting hypernasality among Arabic speaking individuals with velopharyngeal dysfunction.

## 2 MATERIALS AND METHODS

### 2.1 Patient Recruitment

Study participants were six individuals aged from 9 to 61 years (mean age= 39 years) who exhibited hypernasality associated with other speech difficulties including voice, articulation and swallowing disorders. The underlying etiologies of hypernasality included head injury, cerebrovascular accident and neurological disorders. All six participants were fitted with PLA to enhance their hypernasality. The outcome measures included nasalance scores and perceptual speech acceptability ratings. Participants were recruited from the speech pathology and audiology unit patient list of King Abdullah University Hospital (KAUH), Irbid- Jordan between October 2013 and August 2015. Written informed consents were taken from all the adult participants or the guardians of under-aged participants. Endorsement for this project was granted from the Institutional Review Board at KAUH. The inclusion criteria for participation were the presence of hypernasality due to VP impairment, intact hearing function, the presence of at least 2 bilateral premolars and 2 bilateral molars for retaining PLA. Participants with dental implants replacing molars or premolars were excluded from the study.

#### 2.1.1 Case Report 1

A 61 years old man was referred to the audiology and speech pathology center in January 2015 complaining of hypernasality and hoarseness of voice since 2 months. The patient had been referred from the neurology department diagnosed with Amyotrophic Lateral Sclerosis (ALS). Medical history revealed hypertension and hyperlipidemia since 12 years, besides, the patient was healthy otherwise. The patient reported generalized body weakness particularly in both upper extremities 6 months prior to the first diagnosis with ALS. The hearing threshold using pure tone audiometry showed intact hearing function.

#### 2.1.2 Case Report 2

A 31 years old male patient was involved in an automobile accident in August 2013. Multiple fractures ensued including closed-fixed fracture of the base of the skull in the posterior cranial fossa and open fracture of the mandible that warranted immediate total osteoplasty resulted in retrognathism. Speech evaluation revealed flaccid dysarthria with moderately unintelligible speech. The primary speech features presented were hypernasality, nasal emission, breathiness, dysphonia, imprecise consonants, slow rated speech, low loudness level and shortness of breath.

#### 2.1.3 Case Report 3

A 33 years old man suffered a stroke in June 2013. Brain MRI showed multiple small acute infarcts best appreciated on DWI images in the pons and cerebral peduncles associated with ischemic foci. Feeding was done via PEG tube due to aspiration of all food consistencies. One month later, speech evaluation revealed imprecise articulation and slow speaking rate, voice disturbances especially harshness with hypernasality. Pure tone audiometry showed normal hearing threshold. The patient had intact auditory comprehension as well as reading skill. However, the patient showed expressive aphasia, ineligible hand writing with noticeable graphemic paraphasia.

#### 2.1.4 Case Report 4

A 9 years old female had a history of head injury in July 2012 as a result of automobile accident. Medical history showed that the child had severe anarthria. Hypernasality and low loudness level were residual speech difficulties that impacted speech intelligibility. Hearing evaluation was within normal limits. Clinical examination showed difficulty in blowing and whistling. Speech evaluation revealed dysphonia, hypernasality and marked nasal emission especially on pressure consonants. The school record for the patient was poor and the mother reported sub average cognitive functions and slow learning features.

#### 2.1.5 Case Report 5

A 51 years old man was a known case of hypertension since 2005. On June 2014, he went through an automobile accident due to severe headache and blurred vision. Initial speech and language evaluation showed normal auditory comprehension and reading abilities. The patient was encountering severe limitation in speech output and tended to respond by barely intelligible writing. The patient received a speech therapy program; therapy outcomes revealed improved oral communication and legible hand writing in spite of flaccid dysarthria that impacted speech intelligibility. The speech difficulties were slow speech rate, employing imprecise consonants and hypernasality. The patient was reenrolled in another speech therapy, a weekly program for one month based on 6-hour sessions. The therapy outcomes ended up with hypernasality, slow rated speech and moderately intelligible speech. The patient met the criteria for the PLA and was fit on August 2014.

#### 2.1.6 Case Report 6

A 49 years womanwas diagnosed with multiple sclerosis in 2004. She was referred from the neurology department complaining of speech difficulty. The referral report showed periods of remissions lasted maximally 6 months. Speech evaluation was conducted in March 2015 and showed symptoms including harsh voice quality, low pitch, hypernasality and reduced vocal loudness. She had been enrolled in a speech therapy programs several times with instability in the improvement of these symptoms. She was given a further speech therapy program for three weeks of 1 hr session six times weekly with little improvement.

### 2.2 Fabrication of Palatal Lift Appliance

**Fig. (1):**
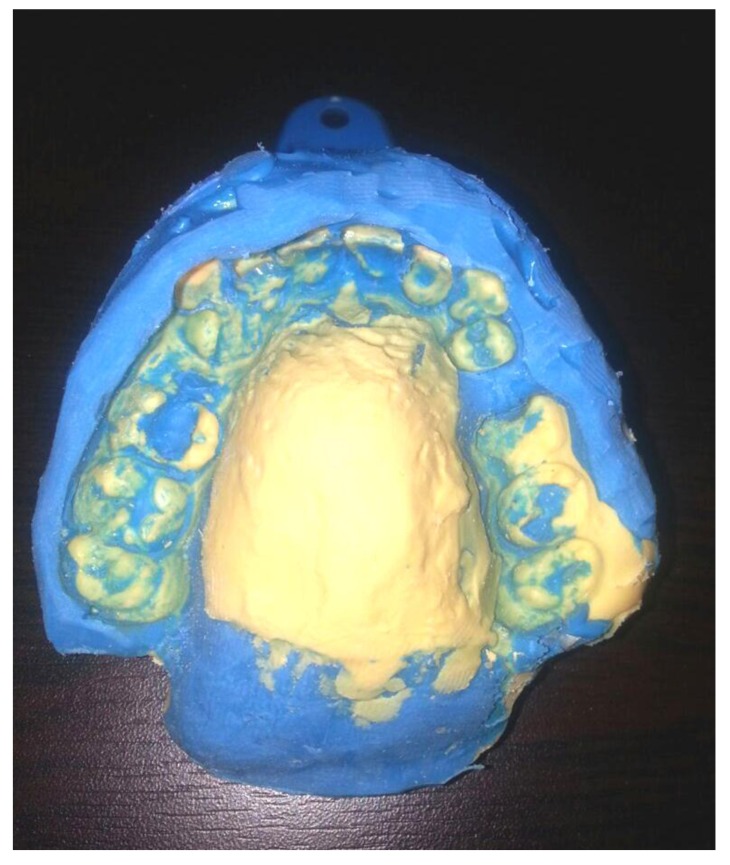
Final impression of maxillary arch extending to the soft palate.

**Fig. (2):**
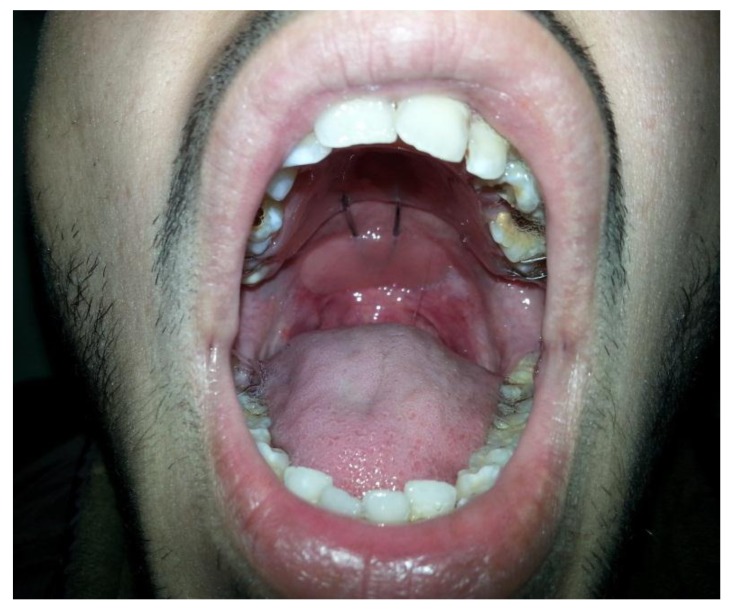
Palatal lift appliance in position.

A posterior extension of stock tray was made using modeling wax (Tenatex Red, Kemdent, UK) in order for soft palate to be superiorly positioned. A preliminary impression was taken using a dental fast setting alginate (Tropicalgin, Zhermack, Italy) with adjusted stock tray. The impression was poured with stone to obtain the preliminary cast on which wax spacer was adapted, and a special tray was constructed using light-curing special tray material (Palatray XL, Heraeus Kulzer GmbH, Germany). The final impression was taken using additional silicone material (Elite HD+, Zhermack, Italy) (Fig. **1**). The impression was poured using type IV gypsum material (Elite rock, Zhermack, Italy). At least two Adam's clasps / C-clasps were fabricated bilaterally on maxillary first molars. The maxillary section of the palatal lift with posterior wire extension (retentive loop for lift pad) was fabricated with the similar method used in partial denture using heat cure acrylic resin (Meliodent Heat Cure, Heraeus Kulzer, Germany). The posterior retentive loop was extended 2cm to the fovea palatina to facilitate supporting for lift pad portion. The loop was fabricated to be in contact with the soft palate and at the same level as the hard palate. Patients were then asked to wear the maxillary portion for 2 weeks to get used to of it. In order to construct the velar portion of the appliance, the loop was tried-in and modified as required to ensure complete seating. Thermoplastic modeling compound was applied to the loop, formed, and heated in warm water to form a soft compound before inserting it into the mouth. Small incremental additions were posteriorly added to the compound until a light contact between the soft palate and posterior pharyngeal walls was achieved. The velar portion of the appliance was replicated in heat cure acrylic resin. Appliance was finished and polished to eliminate any sharp surfaces that might injure the displaced soft palate. The appliance was inserted in the patient's mouth (Fig. **2**) and any required adjustments of clasps were made. The same procedure was followed to construct the PLAs for the six participants in this study.

### 2.3 Appliance Insertion and Method of Evaluation

**Fig. (3):**
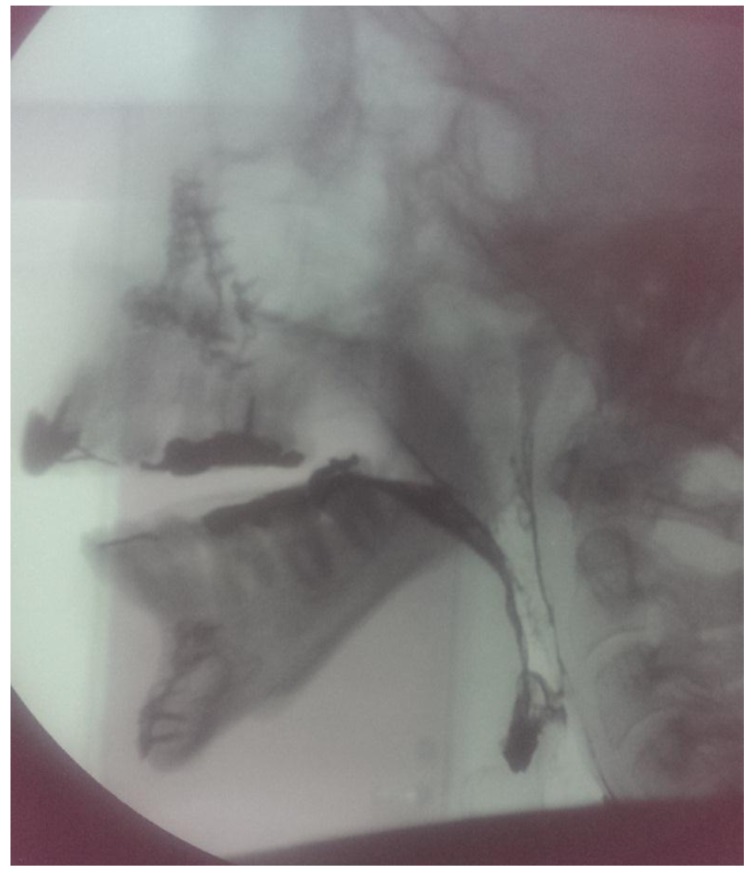
Lateral cephalometric radiograph prior to palatal lift appliance insertion.

**Fig. (4):**
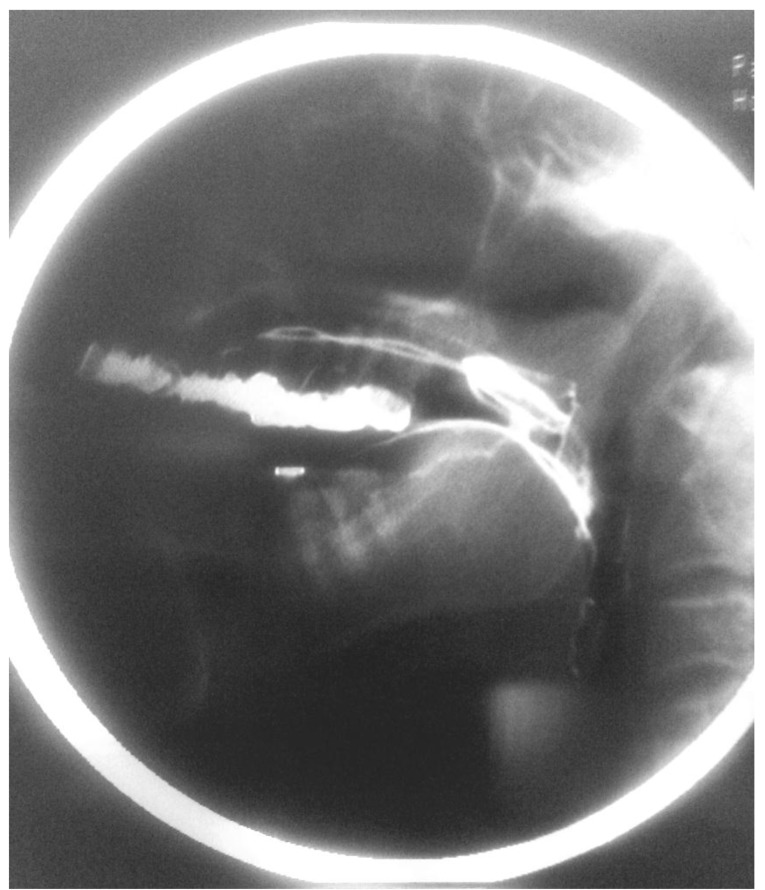
Lateral cephalometric radiographs post palatal lift appliance insertion.

The adequacy of the PLA and its effectiveness in reducing the excess in nasal air flow were confirmed clinically by assuring adequate nasal breathing and lack of dyspnea. Lateral cephalometric radiographs were taken of the patient prior (Fig. **3**) and post PLA insertion (Fig. **4**) to assess the adequacy of palatal lifting. On average, the gap between the superior surface of the PLA and the posterior wall of the pharynx ranged between 2-4 mm. This size of this gap is considered adequate with double function maintenance, *i.e*. speech production and breathing. Other reports didn’t convey the actual size of the velopharyngeal gap, however, a description using small, medium, or large size was denoted [12].

### 2.4 Nasometric Assessment

The patients completed a nasometric assessment in two experimental conditions; with and without the appliance. Nasalance scores were obtained using simplified nasometric assessment procedures (SNAP-R, 2005). Nasalance scores were collected for 8 stimuli (7 orals and 1 nasal). The nasometer II model 6450 (Kay pentax Inc.) was used to record and analyze the nasalance scores of the syllables for the nasometric assessment in the two conditions with and without appliances. The nasometric assessments with appliances were recorded immediately after wearing the appliance.

Based on the previous descriptions of the literature [13, 14], the cutoff value of 27% was used to interpret the nasalance scores, Therefore, nasalance scores values of 27% or below were considered indicative of normal speech resonance, while nasalance scores higher than 27% indicate hypernasality.

### 2.5 Perceptual Rating of Nasality

The normative values of nasalance scores for Jordanian Arabic speakers have not been up to date; therefore, perceptual rating of nasality was used as an alternative to evaluate any change in hypernasality before and after wearing the PLA. For the classification of the degree of nasality, a 4-point equal appearing scale was used, 1 indicating the absence of hypernasality and the scores of 2, 3 and 4 indicating the presence of hypernasality (mild, moderate and severe, respectively). One sentence with excessive number of oral consonants and least nasal consonants was for the purpose. The sentence means in English “The student reads the book”. An expert panel of three speech pathologists with at least six years-experience served as raters. Each member of the panel listened 12 recorded samples (6 with and 6 without PLA) twice in a sound treated room independently and necessitated to evaluate the degree of nasality. The raters were blinded to PLA condition during perceptual rating. The speech samples were presented two times and randomized to control for order effect for the analysis of intra- and inter-raters agreement.

### 2.6 Statistical Analysis

Descriptive statistics were used to find the means for each nasal subset for both with-PLA and without-PLA conditions. A paired Student's t test was carried out to compare the mean nasalance scores in both conditions. The inter- and intra- rater agreement of the perceptual ratings of nasality was calculated to detect the percentage of agreement among the raters and reliability of each rater in both situations (with and without PLA).

## 3 RESULTS

Distribution of the participants’ demographics and frequency of visits required for the customization and fitting of the PLA are depicted in Table **1**.

Amyotrophic Lateral Sclerosis (ALS), Traumatic Brain Injury (TBI), Cerebrovascular Accident (CVA), Multiple Sclerosis (MS).

Speech assessment showed marked reduction in hypernasality as revealed by the significant reduction in nasalance scores *Pa, Pi, Ma, Mi, a,* and *i* (*P*<0.05). The scores of the subtests *u* and *m* were not significantly affected by the insertion of PLA. The means for Nasalance scores (NS) of the syllable repetition and sound prolongation subtests are listed in Table **2**.

The perceptual ratings of the six patients with and without PLA are summarized in Table **3**. The average rating of the six patients showed a decrease in hypernasality from 3.55 to 1.17. The first trial ratings were used to calculate the inter-rater agreement. Inter-rater agreement revealed 78% among the three raters. Intra-rater agreements for 1^st^ rater, 2^nd^ rater and 3^rd^ rater were 83%, 83%, and 92% respectively.

## 4 DISCUSSION

Velopharyngeal incompetence is coupled with wide range of speech disorders including hypernasality, reduced intraoral pressure and compromised articulation. In such cases, prosthetic management may be considered the best treatment choice [15]. This study involved an interdisciplinary team including prosthodontist, speech pathologist and dental technologist and aimed to investigate the effect of PLA on the hypernasality of VP dysfunction patients. The results showed a reduction in hypernasality on insertion of PLA. These findings are in agreement with the previous studies [16-19].

In this study, the nasal consonant /m/ had not been significantly affected after the insertion of the palatal appliance. Normally the /m/ is produced while the velopharyngeal valve is open, therefore, intra oral pressure is not necessary. The insertion of the PLA didn’t attempt to close the velopharyngeal valve completely, *i.e*. the gap ranged between 3-6 mm and therefore there was no need to lift the palate during the articulation of this sound. On the other hand, other test stimuli such as the vowels /a, u, i/ and the oral syllables /Pa, Pi/ obligate the presence of intra oral pressure. In partial, the /Ma, Mi/ syllables are a sequence of nasal-oral syllables which are produced in the oral and nasal cavities and need certain oral pressure. This justifies positive improvement in producing these speech sounds after the palatal lift insertion. The mechanical obstruction of the flow of air to the nasal cavity increased the oral pressure and improved the balance between the oro-nasal combinations of resonance, therefore; helped recovering the speech orality. These findings are also in agreement with previous studies [20, 21].

The perceptual rating showed that hypernasality was reduced in all patients from moderate to severe hypernasality to mild or absence of hypernasality. The perceptual rating and nasometric measurements were taken after immediate insertion of PLAs in order to highlight the immediate improvement that can be achieved using this appliance. Therefore, no attempt was made to examine the effect of PLA maintenance for extended period of time on reduced hypernasality. Several studies showed that wearing PLA for sufficient period of time may cause a positively alteration in the neuromuscular response of the patient's velopharyngeal valve, which in turn improves function and reduces disuse atrophy [22, 23].

A combination of nasometric measurements and perceptual ratings was used to provide objective and subjective assessment, however, a correlation between nasalance scores and the perceptual rating of nasality was not tested in this study due to limited number of patients, and different stimuli used for these measuring tools which could affect the correlation. Some previous studies, which investigated the correlation between nasalance scores and the perceptual rating, showed high correlation between nasal scores and perceptual rating of hypernasality with the use of oral stimulus [24-26], while others reported low correlations [27, 28]. This wide variation in the results of previous studies can be explained by the use of different methodologies between studies, including differences in the language, speech stimuli, dialect, and the scales used for perceptual ratings [29, 30]. The degree of knowledge and experience of the rater to the perceptual ratings can also attribute to the variations between the studies [31].

**Fig. (5):**
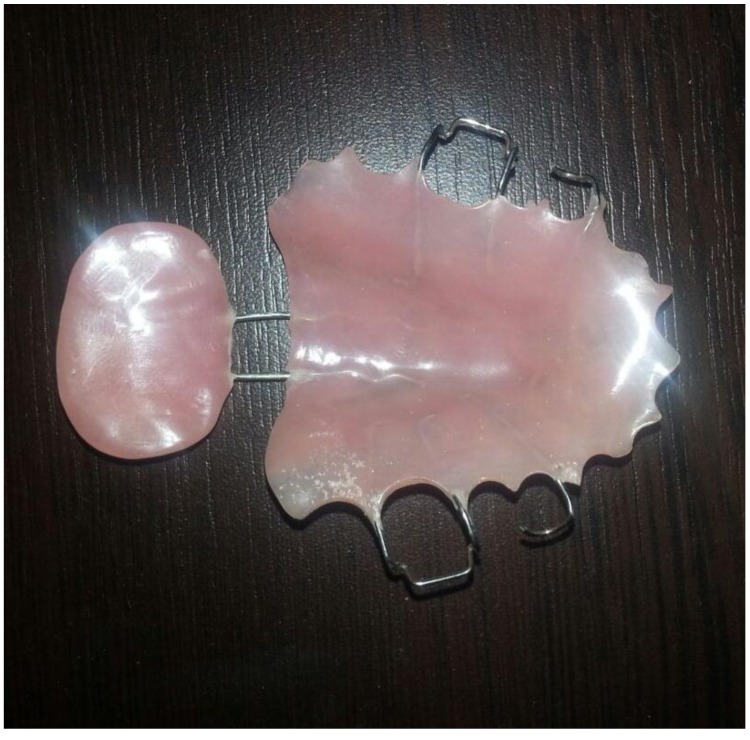
Palatal lift appliance.

PLA can be made out of a metal alloy and acrylic combination or entirely out of acrylic resin. In this study, the use of an interim acrylic (metal free) PLA (Fig. **5**) rather than cast metal framework was because the patient acceptance, for the cast metal palatal lift, was usually low. This is due to the discomfort caused by the strong lifting force exerted by the lift portion of the cast metal palatal lift. An interim PLA can allow the patient to get adapted to the palatal extension gradually. The force applied on the soft palate by the PLA can be gradually increased by elevating the velar portion of the PLA on multiple visits [15].

The success of PLA depends on the available number of maxillary teeth that can provide the required retention for the appliance [32]. In the present study, at least 2 wrought-wire clasps (Adams clasps/C-clasps) have been constructed bilaterally to provide a simple and relatively inexpensive method of retention of the appliance to overcome the downward force of the palatal musculature. The selection of abutment teeth, to retain the PLA, should be carefully evaluated in order to give maximum advantage to the lift. Posterior teeth act as the most appropriate abutments because they are closer to the cantilever extension [20]. Therefore, maxillary 1^st^ molars were chosen to receive Adam's clasps or C-clasps for all cases participated in this study. For additional retention, C-clasps were constructed for the maxillary 1^st^ or 2^nd^ premolars.

## CONCLUSION

Although this study was preliminary in nature, it showed that prosthetic management can be a useful tool of intervention to treat hypernasality of patients with velopharyngeal impairment. It also demonstrated the significance of interdisciplinary involvement in the care of people with velopharyngeal incompetence.

## LIMITATIONS AND FUTURE RESEARCH DIRECTIONS

The limitations of this study may include; first, this study was preliminary and data analyzed are considered as exploratory in nature; therefore, caution needs to be considered when generalizing the findings. Second, the patients were heterogeneous in terms of age, gender, and etiology of velopharyngeal impairment. In future studies, using a larger sample size of participants is considered necessary to increase the confidence and generalization of the clinical outcomes. Also further studies are needed to investigate the effect of PLA on hypernasality and speech intelligibility among patients with VPI pre and post speech intervention, and to examine the effect of PLA over an extended period of time.

## Figures and Tables

**Table 1:** Distribution of the demographics of participants.

Participant	Age	Gender	Diagnosis	Pre-existing Prosthesis
1	61	M	ALS	Yes
2	31	M	TBI	No
3	33	M	CVA	No
4	9	F	TBI	No
5	51	M	CVA	No
6	49	F	MS	No

**Table 2:** Distribution of the individual with their mean nasalance scores (%) for the syllable repetition and vowel prolongation tasks.

**Patient**	**1**	**2**	**3**	**4**	**5**	**6**	**Mean (SD)**	**Hypernasality**	***p*-value**
**Pa-Pre**	48	22	22	42	33	18	30.8 (12.2)	Hypernasal	0.008*
**Pa-Post**	26	5	17	30	21	14	18.8 (7.9)	Normal resonance	
**Pi-Pre**	57	37	31	42	27	33	37.8 (11.8)	Hypernasal	0.003*
**Pi-Post**	34	20	23	32	18	23	25 (6.2)	Normal resonance	
**Ma-Pre**	68	66	57	45	68	58	60.3 (5.5)	Hypernasal	0.008*
**Ma-Post**	38	50	39	27	23	49	37.6 (10.9)	Hypernasal	
**Mi-Pre**	69	89	82	47	75	85	74.5 (8)	Hypernasal	0.028*
**Mi-Post**	42	75	61	40	25	80	53.8 (23)	Hypernasal	
**a-Pre**	27	12	19	20	12	50	23.3 (15.8)	Normal resonance	0.007*
**a-Post**	22	3	14	14	9	36	16.3 (12.8)	Normal resonance	
**i-Pre**	32	31	56	38	19	78	42.3 (23.6)	Hypernasal	0.008*
**i-Post**	22	22	33	27	12	51	27.8 (14.9)	Normal resonance	
**u-Pre**	24	15	17	22	23	61	27 (18.8)	Normal resonance	0.056
**u-Post**	19	7	12	16	17	31	17 (9)	Normal resonance	
**m-Pre**	94	97	93	94	94	97	94.8 (1.9)	Nasal consonant	0.419
**m-Post**	92	95	94	97	92	94	94 (1.3)	Nasal consonant	

* significant.

**Table 3:** Perceptual speech ratings for the six patients with and without palatal lift appliance (PLA).

**Patient**	**Trial**	**Rater 1**		**Rater 2**		**Rater 3**	
		**With PLA**	**Without PLA**	**With PLA**	**Without PLA**	**With PLA**	**Without PLA**
1	Trial 1	1	3	1	3	1	3
	Trial 2	2	3	1	3	1	3
2	Trial 1	1	3	2	3	1	4
	Trial 2	1	3	1	3	1	4
3	Trial 1	1	3	1	3	1	3
	Trial 2	1	4	1	3	1	3
4	Trial 1	1	4	1	4	1	4
	Trial 2	1	4	1	3	1	4
5	Trial 1	1	4	1	4	1	3
	Trial 2	1	4	1	4	2	3
6	Trial 1	2	4	1	4	2	4
	Trial 2	2	4	1	4	2	4

Absence of hypernasality (1), mild hypernasality (2), moderate hypernasality (3), and severe hypernasality (4).
